# Leveraging Food-Related Values for Impact in Community Nutrition Education Programs (Interventions)

**DOI:** 10.3390/foods12040714

**Published:** 2023-02-07

**Authors:** Terrence Thomas, Cihat Gunden, Befikadu Legesse

**Affiliations:** 1 Department of Agribusiness, Applied Economics and Agrisciene Education, North Carolina A&T State University, 1601 E. Market St., Greensboro, NC 27411, USA; 2 Department of Agricultural Economics, School of Agriculture, Ege University, Bornova, 35040 Izmir, Turkey; 3 Smart-Eco Consulting, Silver Spring, MD 20906, USA

**Keywords:** food-related values, food-related behaviors, nutrition programs, intervention, multivariate analysis

## Abstract

This study draws attention to the potential benefits of leveraging food values to amplify the impact of nutrition education programs. The study has collected data via a telephone survey from 417 randomly selected residents in Guilford County in the state of North Carolina. In our analysis, we have identified and used three underlying dimensions (ethical, social environmental and sensory) that summarize and capture the meaning of food-related values instead of a list of food values commonly used in the literature. Researchers have then used these dimensions as clustering variables to produce three segments from the data: value-positive, value-negative, and hedonic. Results show that residents in the value positive segment had positive perceptions of all values, those in value negative segment had negative perception of all values, and those in the hedonic segment had only positive perception of sensory values. A key finding is that value-positive residents have healthier food-related lifestyles and food-related behaviors than residents in the other segments. Interventions should focus on value-negative and hedonic residents and emphasize value-based education tailored to strengthening social/environmental and ethical food values. To ensure success, interventions should graft healthier lifestyle habits and behaviors on familiar behaviors and lifestyle.

## 1. Introduction

Guilford County is a food-insecure county in North Carolina, USA. A total of 26 census tracts were designated as a food desert (FD) in 2018, up from 24 in 2010 [1]. In the literature, the typical features that define a food desert are location in underserved, low-income areas with reduced access to transportation, lack of full-service supermarkets, and a prevalence of convenience stores [2]. An area designated as a food desert represents an extreme case of food insecurity. Access to food in these areas is typically limited to low quality and preserved food items sold in convenience stores. Over time, residents of food deserts adapt to this deficient food environment. The effect of this adaptation is reflected in their eating habits and, consequently, their health status [3,4,5,6]. However, there are studies such as [7,8] that find no relationship between the food environment and health status of residents. Nevertheless, there are many other studies such as [9,10,11,12,13,14], which support the belief that the defining features of FD contribute to disparities in diet and adverse diet-related health outcomes. Additionally, [15,16,17] draw attention to the effect of the wider social environment through social network effects. In addition, as [17] points out, exposure to the example behavior of peers in one’s surroundings have a powerful influence on health behaviors and health outcomes. Therefore, there is significant evidence from diverse streams of the literature which support the view that FD as well as NFD residents acquire and interpret knowledge about food and healthy eating behaviors from their surroundings. As a result, people behave in ways that are shaped by their extant environment. Simply put, behavior is shaped in context [18,19,20]. Further, we note that North Carolina is rated as the 12th hungriest state in the nation [21], and Guilford County has approximately 53,000 residents who are food insecure and are at risk of developing diet-related diseases. And although the number of communities in America without sufficient access to affordable healthy food has declined, there is still concern about the racial and economic disparities in food access [22].

In response to this situation, the city of Greensboro and the county have supported the development of urban farms and community gardens to increase access to healthy food. More generally, and just as important, is the observation that most Americans do not meet the Dietary Guidelines for Americans (DGA). These guidelines assist Americans in selecting foods that will supply the required nutrients, promote health, support active lives, and prevent chronic diseases. These guidelines highlight the importance of making choices to better support healthy eating patterns [23]. Even with these programs in place, Ref. [24] reports that the diets of most Americans usually fall short of meeting the federal dietary recommendations for whole grains, fruits, nuts, and legumes, with disparities in dietary quality across income levels.

Policy makers at the national and local levels responding to the reported impact of poor diets and the perceived lack of access have developed measures to eradicate food deserts and improve access to nutritious food for everyone. These measures focus more on improving access to fresh fruits, vegetables, and other healthy foods. For example, there are many initiatives at the local, state, and national levels designed to tackle the problems of food access, food insecurity, and address lifestyle issues related to unhealthy eating habits. At the national level, there are programs that include child nutrition programs, the Supplemental Nutrition Assistance Program (SNAP), and emergency food assistance, among many other programs [25]. In addition, Ref. [26] is responsible for developing and promoting dietary guidance that links the best evidence-based scientific research to the nutrition needs of Americans. At the local level, in the study area, the Renaissance Cooperative Grocery store, established by a coalition of city, community, and business leaders, provided residents of Eastern Greensboro with access to a wider range of healthy offerings. However, it closed its doors after a very brief period of operation due to low levels of patronage [27], which may be indicative that access may not be very important.

Despite these programs and the many commercial diet and exercise programs promoted in the media, nutrition-related diseases continue to be among the leading cause of death in the U.S., and more than two-thirds of Americans are obese. Obesity is a chronic disease and a risk factor for nutrition-related diseases, such as heart disease, cerebrovascular disease, and diabetes, which is cause for concern. However, what is most concerning about this situation is the lack of evidence indicating a decrease in or even a leveling-off of obesity rates [28].

Thus, improving access by itself may not result in residents increasing their purchasing of healthier offerings [29,30]. What is going on here? An increasing number of scholars, for example [31], argue that social science models often display none or very little predictive capacity because the underlying generative mechanism of social phenomena is not stable, so these models may only capture instances of phenomena as they emerge in a particular context and may not be stable over time and context to be generalizable. The implications are that it may be more useful to work with phenomena as it is expressed in a particular context and be willing to learn and adjust iteratively. Additionally, the food and diet-related behaviors observed across groups and in the population in general are the result of interactions among several interdependent variables. Accordingly, a systems approach to addressing food security seems more useful. Thus, from a systems perspective, reducing the impact of diet-related disease and food insecurity requires considering multiple variables, not just access. It also involves the relationship of healthy communities and societies to sustainable development; that is, ensuring sustainability in consumption as well as in food production is inevitable if society is to achieve the goal of being sustainable [31,32,33].

To clarify the conceptual link between health consciousness and food consumption, it is useful to examine the multidimensional structure of sustainability. The underlying dimensions of sustainability, which is an abstract construct, are conceptualized as temporal dimension regarding environmental concerns (trade-offs between present and future), and social dimension, dealing with ethical concerns (trade-offs between consumers and others) [34]. Sustainable consumption refers to the decision-making that considers the social responsibility of the consumer [35,36]. However, a sustainable (or reflexive) consumer is not an ethical consumer. A sustainable consumer is surrounded by general cultural norms associated with the environment. On the other hand, ethical consumer incorporates social and ethical issues, and feels responsibility for both the environment and society [34,36]. Health and sustainable development make important contributions to each other. An unhealthy society or community is not sustainable. Therefore, health is included in sustainability as the fourth dimension, and sustainable development now consists of environmental, economic, social, and health sustainability. To better understand the relationship between health and sustainability, researchers have suggested the duality of thought [37,38,39]. The duality of health and sustainability refers to the mutual linkages between health promotion and sustainable development that must be considered to produce, reproduce, and constrain each other in a dynamic symbiosis to achieve the goal of a sustainable society. Consequently, in addition to access, policy makers should take into account the food values of insecure communities—the interaction with food that enables a realization of a desirable social or personal goal—which are closely related to environmental and ethical food consumption and the general goal of a sustainable society.

In complex systems, it is counterproductive to work with or try to optimize a single variable to meet an objective. Complex systems continually adapt to change so that interventions that attempt to optimize one feature of the system in isolation from the rest of the system will likely not produce the desired result over the long haul, and the system may adapt to this intervention in undesirable ways [40]. This perspective on attempts to adjust complex systems applies equally well to efforts designed to improve food security outcomes by focusing on a single variable, such as increasing access to healthy food. As [41] points out, a better way to work with complex systems is to identify and address key drivers of the system in tandem. In social systems and in social decision making, underlying generative features such as thoughts, beliefs, and goals (values), produced from interaction with the food system, are key drivers; they are the generative processes that produce the ultimate observable outcomes, such as desirable habits and behavior [32]. In addition, as [41] notes, an individual’s behavior is formed through values that have a position in the center of self-conception. Thus, values are a primary driver of observable behavior. Consequently, this study seeks to investigate the significance of food-related values (frv) in designing intervention programs focused on improving the food security status of Guilford County residents and, consequently, the long run health status of community members, the food system, and environmental sustainability. 

### Literature Review

To explore consumers’ values, attitudes, behavioral intentions, and actual purchase behavior, numerous research studies have employed the theory of reasoned action (TRA) by [42], which includes attitude and social norms, and the theory of planned behavior (TPB) by [43,44] considers perceived behavioral control as well. Ref. [45] utilizes TRA for exploring motivations such as environmental concern, health consciousness, familiarity, etc., on organic attitudes, intentions, and behavior. They found a relationship between environmental concern and organic attitude; health conscious and purchase intention; familiarity and organic purchase behavior. Ref. [46] supports the framework of TPB in predicting purchase intention of organic foods. On the other hand, [47] reveals that different levels of value orientation produces different strengths of TPB along with consumer confidence and values in purchasing sustainable dairy products. Ref. [48] suggests applying the extended TPB that provides a mediating role for environmental concern, which has a higher predictability than TPB and TRA models, to analyze the purchase intention of green products.

Values have been examined in the studies that attempt to find a relationship between global or personal values and consumers’ purchase behavior for a certain group of food products. Ref. [49] investigates the relationship between consumption of organic food and global values, suggested by the value theory in [41]: security, hedonism, universalism, benevolence, stimulation, self-direction, and conformity. The authors acknowledge the positive effects of these values on organic food consumption. In addition, consumers with traditional values are more inclined to buy sustainable products than consumers who are power seekers [47]. In addition, researchers have discovered the remarkable influence of moral attitude and subjective norms on consumers’ willingness to buy organic food [46]. Similarly, Ref. [50] shows that positive attitudes of consumers toward environmental protection are one of the major facilitators of green food purchases.

As for personal values, Ref. [51] explores two different groups based on the List of Values (LOV) developed by [52]: internal and external values. The authors reveal that people who give more importance to internal values (self-fulfillment, fun and enjoyment in life, sense of accomplishment, and self-respect) and less importance to external values (sense of belonging, being well-respected, and security) tend to purchase natural foods. Additionally, researchers have investigated correlations among personal values, attitudes, and behaviors. They have found that excitement has a significant positive correlation with a pro-snacking attitude, whereas a warm relationship with others has a negative correlation [53]. Moreover, self-respect as internal values and security as external values are correlated negatively with convenient food consumption. In contrast, achievement is correlated positively with convenience food product usage, convenience orientation towards food shopping, meal consumption, and meal preparation [54].

In another pioneering study, Ref. [55] has constructed a model that shows a value-lifestyle-behavior relationship. They have proposed a hierarchical structure using the List of Values (LOV) as personal values, a food-related lifestyle instrument developed by [56] as a measure of lifestyle-specific area of food consumption, and food-related behavior [55] as an indication of consumers’ shopping, cooking, and eating behaviors. These research studies show that food-related lifestyle is a mediator between values and behavior [55,56,57,58].

The strength of personal values may be low in explaining consumers’ food-related behavior due to other influencing factors. Food values can be powerful depending on consumers’ prioritization tendencies [59]. The relationship between consumers’ food values and behaviors has been rarely investigated in the literature. In previous studies, consumers were given a certain number of food values related to food consumption patterns [60,61]. These studies attempted to replicate the values proposed by [41,60] the list of food values, including 11 food values based on human values and preferences to determine how these values affected consumers’ preferences for organic food. They employed best-worst scaling and econometric methods in a study in the USA. The authors found that safety, nutrition, taste, and price were the most important, whereas environment, fairness, tradition, and origin were the least important food values. They pointed out the significant influence of food values on consumers’ preferences. Ref. [60] classifies the list of food values into three groups of attributes: credence (naturalness, safety, environmental impact, origin, fairness, nutrition, and tradition), experience (taste, convenience, and appearance), and search (price). Unlike the study conducted by [60], the authors have modified the list by including novelty and animal welfare and excluding tradition. Another definition of food value is the food consumption value (FCV) introduced by [62]. The FCV comprises two relevant values: product value, which refers to physical product attributes, and process values, which is related to practices and characteristics of the production process. The list of food values proposed by [63] and the FCV are directly related as follows: product value is associated with six food values (i.e., taste, price, safety, convenience, nutrition, and appearance), while process value corresponds to the remaining five food values (i.e., naturalness, tradition, origin, fairness, and environmental impact) [62]. As an example of the value-attitude-behavior chain, Ref. [64] investigated the impact of food-related values on consumers’ food purchase behavior along with the mediating role of consumer attitudes toward eight food product categories. They have found a partial mediation of values through attitudes, meaning that food values have a direct impact on attitudes, and attitudes influence food purchase behavior. Recently, Refs. [65,66] have considered food-specific values developed by [60] instead of the [55] model that utilized personal values, defined as LOV above, and have explored the influence of food values and food-related lifestyle on food-related behavior among food desert residents in the US. They have found that self-centered consumers tended to eat fast food.

Consumers have recently increased their interest in ethical, environmental, and health issues. Since there is a relationship between healthy eating and environmental and ethical consumption, this interest of consumers has changed their preferences [34,63]. Health-conscious consumers are concerned about their personal health and well-being. Therefore, they pay more attention to improving their health and quality of life. In addition, they are more likely to select healthy food options and are concerned with eating healthier [67,68]. For instance, high health-conscious consumers cook regularly and plan healthier meals with whole grains, fruits, and vegetables more than low health-conscious consumers [69]. Similarly, Ref. [67] applies the Health Belief Model to predict consumers’ behavioral intentions and has found that health-conscious consumers are influenced by the availability of healthy food at full-service restaurants. There is a wide breadth of literature that draws attention to the role of values in our interaction with food. More recently, this includes [70], an investigation of the role culture; Ref. [71], food values and heterogeneous consumer responses to nanotechnology; Ref. [72], examining food purchase behavior and food values during the COVID-19 pandemic; Ref. [73], comparison of food values for consumers’ preferences on imported fruits and vegetables within Japan, Taiwan, and Indonesia; Ref. [74], beliefs, values, and sociocultural patterns related to food safety in low- and middle-income countries: a synthesis of the descriptive ethnographic literature; and Ref. [75], food values drive Chinese consumers’ demand for meat and milk substitutes. These studies demonstrate the influence of values on varying aspects of food-related behavior in a variety of contexts, suggesting the potential efficacy of values as leverage for modifying behavior. 

In summary, in the hierarchical structure proposed by [56], personal values are abstract top-level goals in a hierarchical cognitive structure. On the bottom rung of the hierarchy are specific product perceptions that are situation-specific. The lifestyle construct is defined as an intervening cognitive structure that links abstract personal values to situation-specific product perceptions. Ref. [55] points out that the hierarchical cognitive structure is used to process information through a bottom-up and a top-down route. They argue that the bottom-up route is triggered by external input from product perception—derived from the interaction with food —that is processed through the hierarchy to activate personal values. Based on these differences among individuals, it is also possible to divide or segment Guilford County residents using the clustering dimensions that summarize food-related values and describe the predisposition of residents toward food and the food system based on their classification into segments, thus making it possible to design intervention programs that address the education and other support needs of segments based on segment-specific behavior.

## 2. Methodology

### 2.1. Sample Size

This study was reviewed by the North Carolina A&T State University IRB and was determined to be exempt from further review according to the regulatory category cited above under 45 CFR 46.101(b). The study used [76] sample size formula to determine an appropriate sample size. The sampling protocol was sent to Survey Sampling International, Six Research Drive, Shelton, CT, USA. They drew a random sample from the population of the county. Ten thousand telephone numbers were drawn, allocated equally among cell phones and landline numbers and considering non-working telephone numbers and businesses. Zip-code identifiers were associated with each telephone number, which enabled the identification of FD residents from NFD residents. Trained enumerators collected data via telephone survey, using the Survey Monkey platform to record responses from a random sample of 417 respondents. After cleaning the data, the analyses used 352 respondents.

### 2.2. Content of Analyses

Theoretically, we considered value-lifestyle-behavior relationships proposed by [55]. In this context, the content of analyses employed are presented below:

(1) Factor analysis was conducted to determine the underlying dimensions that summarize and capture the meaning of food values generally accepted by the society. The dimensions were labeled depending on the definition of food values in the literature.

(2) The factor scores of the three dimensions of food values obtained from the factor analysis were used as variables to divide the consumers into groups in the cluster analysis. These scores are the composite measure generated for each observation in each factor derived from the factor analysis. Considering differences in terms of the perception of food values, consumer profiles in each group were defined by using dominant dimensions. A statistical comparison was made among the consumer segments based on the three dimensions used as clustering variables. Then, consumer groups were labeled.

(3) The statements that identify food-related lifestyle were used to make a comparison between the groups of residents defined by segments that were obtained from cluster analysis. This comparison enabled us to determine the effect of food values on food-related lifestyle.

(4) Likewise, statements that identify food-related behavior were used to make comparisons between groups of residents that were obtained from cluster analysis to identify differences in food-based behavior between the segments.

(5) Researchers generally produce a list of food values that capture the major values describing food consumption [60]. However, the present study employs a different approach. We initially considered the food values used in the previous studies and then performed a multivariate analysis technique for identifying the underlying values that influence food-related lifestyle and behavior. Thus, the approach determined whether the values could measure consumers’ food values or not in the study area, instead of measuring solely the importance of pre-determined food values assumed to be the values that influence consumers’ food-related lifestyle and food-related behaviors.

### 2.3. Analytical Framework

The framework we used to derive the dimensions from exploratory factor analysis is given below:

Factor analysis was conducted using IBM SPSS Statistics 25 to determine the underlying dimensions that summarize and capture the meaning of food values to make these values comparable and understandable in terms of their influences on food-related lifestyle and food-related behaviors [66]. Factor analysis enabled us to reduce the number of values by combining two or more values into a single food value dimension. Eventually, the analysis represented food values by their common underlying dimensions. In the present study, principal component analysis utilizing varimax rotation was performed to determine the minimum number of dimensions that will account for maximum variance in the data [77,78,79]. Factor analysis process described by [65,66] was followed as outlined in the steps given below:

Initially, factor analysis was applied using 11 food values and 352 responses. In the preliminary result, all food values were checked to ensure that they met the requirements for employing factor analysis. Then, three problematic food values were removed from the analysis due to low communalities (variance problem) and a complex structure problem (uni-dimentionality problem) [79], meaning that safety, convenience, and price do not capture adequately the meaning of food values.

Cluster analysis was used to segment consumers into groups based on food-related values. The aim of cluster analysis is to classify consumers into relatively homogeneous groups called clusters, such that each cluster is as homogeneous as possible with respect to the clustering variables. Furthermore, it is a useful classification technique for respondent profiling [78,80]. The present study employed hierarchical clustering. Ward’s method, which minimizes the sum of squares of any two clusters that can be formed at each step, was chosen as the clustering method to evaluate the distance between clusters [77,79,81].

Point Score Analysis was conducted to rank food values by the importance consumers put on each value. Food values were ranked considering the share of summed scores reported by consumers in total scores (10 × 352) for each value.

## 3. Results and Discussion

Table 1 shows food values used in this study with their description [60] and corresponding attributes [61].

Table 1 shows basic descriptive statistics on food values used in this study. A reliability test yielded a Cronbach’s alpha of 0.78, meaning that all the items included in the analysis were measured with a reasonable degree of reliability. The Friedman test, which is significant (χ^2^ = 867.451; *p* < 0.01), confirms that the degrees of importance reported by residents for food values are significantly different. The results indicate that safety is the most important food value, followed by taste, nutrition, and appearance. The least important ones are tradition, fairness, convenience, and origin. İt is unsurprising that residents accord more value to safety, taste, nutrition, and appearance in that order, given the salience of these values to residents.

That is, residents can easily associate these attributes with their everyday lived experience with food even though the relative value of these attributes may vary depending on the context. Table 2 shows the results of a sample of research studies in the literature that rank food values as the most important, intermediate, and the least important. The data in Table 2 lends support to the relative salience of values to people across contexts. The absence of price from the top four values in this study is a surprise finding. We note earlier the role of the environment [9,10,11,12,13,14,15,16,17] in shaping behavior and values. Given this background, we can infer that the high impact value (82.9) of environmental factors influence the expression of other food values. For instance, we propose that the environment affects physical access to healthy food—the lack of full-service supermarkets and the preponderance of convenience stores. Environmental factors shape tradition and fairness (ethical values) through exposure, social learning, and network effects [17]. In this regard, the social advocacy (a combination of social learning, engagement, and exposure) of the community-based organization, Citizens for Economic and Environmental Justice, creates a strong sense of environmental quality and equity among residents that provides robust community support for addressing issues related to the landfill in their community. Furthermore, the social environment is central to the culture and transmission of traditions, which include taste.

Following factor analysis, three problematic food values were removed from the analysis due to low communalities and complex structure problems [79]. These were safety (communality less than 0.5), convenience (uni-dimensionality, loading higher than 0.4 on more than one component) and price (communality less than 0.5). This meant that safety, convenience, and price did not capture adequately the meaning of food values. Factor analysis was repeated using the food values retained.

Employing the Kaiser Rule [81], researchers selected three dimensions with eigenvalue >1 as shown in Table 3 for use in further analysis. The total variance explained was 64.55%. The Kaiser-Meyer-Olkin (KMO) measure of sampling adequacy was 0.74, indicating factor analysis was appropriate [79]. Bartlett’s test of sphericity was significant (*p* < 0.001), meaning that the correlation matrix is significantly different from identity matrix [78]. The reliability test conducted to assess the internal consistency among the set of values on the dimension produced a Cronbach’s alpha of 0.74. Finally, the dimensions entitled ethical, social, and environmental, and sensory (emotional) values were identified as the underlying dimensions that summarize residents’ food values in the sample from the study area.

As shown in Table 4, the underlying dimensions of food values derived from factor analysis were compared based on the level of importance residents assigned each dimension. The Friedman test showed a statistically significant difference among the dimensions, which means that sensory (emotional) values followed by social and environmental values were more important than ethical values in the study area.

The differences among the three dimensions indicate that program designers and policy makers should pay attention to the food values, which are more salient to residents. For example, nutrition education programs should emphasize designing tasty and attractive dishes that incorporate fruits and vegetables using current popular staples as a foundation, while taking the opportunity to point out the environmental and nutritional benefits. The relative importance of the social environmental value indicates residents becoming increasingly aware of the environmental impact of their food choices.

Table 5 presents the consumer segments defined by factor scores derived from cluster analysis. Three distinct segments of consumers were obtained with respect to the dimensions. Table 6 presents the number and percentage of respondents in each segment in the last two rows. Positive and negative factor scores are presented for each segment (Table 6). Residents with positive scores indicate a positive perception of the food value dimensions, while the residents with negative scores have a negative perception of the dimension. Segment I represents 47.70% of residents, and they have a positive perception of all food values. Segment II represents 18.80% of the residents, and they have a negative perception of all dimensions of food values. Segment III represents 33.50% of the respondents, and they have a favorable view of sensory values, but unfavorable views of ethical, social, and environmental values. Considering differences in the perceptions of food values, residents’ profile in each segment was defined using the dominant dimensions in a segment. For example, in segment I, where residents have a positive view of all values and the dominant values are positive, this segment is labeled value-positive. Segment II is labeled value-negative since all food values are perceived as negative, and segment III is labeled as hedonic since the dominant food value is sensory with positive signs.

Comparing the segments based on demographic variables may provide an enriched interpretation of residents’ profiles (Table 6). There are no significant statistical differences among the segments in terms of income. However, there are significant statistical differences among the segments in terms of respondents’ age and education. Segment I is statistically different from segment II and segment III. Residents in segment I are older than consumers in segment II and segment III. Also, segment I has a relatively lower education level than the other two segments. To sum up, we can conclude that value-positive consumers are older and lower-educated. The detail number and percentage of demographic variables are presented in Table 7.

The demographic data in Table 7 above indicate that in the total sample, most respondents are female (63.42%) and are older than 56 years (58.57%). A similar patten holds for food desert and non-food desert respondents. A little less than half (approximately 47.7%) fall below the federal poverty level, defined as income below 200% of the stipulated level. For a household of three people (the average household size for residents of the food desert is 2.37), this is $46,060.00 [82]. Most respondents have better than a high school education (52.28%) and just about 52.28% are employed full time. Age and income level of respondents in the sample suggest a plausible explanation for the importance of taste as a food value, which is, before everything else, older individuals with limited income ensuring that their food satisfies their taste.

Table 8 shows residents’ food values by gender. One of the most important results obtained from this study is that women perceive all food values as positive, and men perceive food values as negative. This finding may explain the very low participation of men in our nutritional education classes we conducted from 2017 through 2020. Only two or three men out of a group of 12 participants attended workshops each week over a period of 8 weeks. It is also the case that most participants are older women, which is consistent with the findings of Table 8.

As shown in Table 9, food desert residents perceive ethical values as positive, whereas non-food desert residents perceive social-environmental and sensory values as positive. One plausible explanation for this difference is that FD residents are primarily in low-income underserved communities with relatively high unemployment rates and impoverished food environment, which may make ethical values more salient to them compared to social/environmental and sensory values.

Table 10 presents data that shows FD and NFD are not useful for differentiating among the segments in this sample from the study area (Chi-Square: 1.710; Sig.: 0.425). This finding means that the values provide no basis for treating FD differently from NFD based on food values as defined in the study. We can also infer that the value segments would tend to influence behavior and lifestyle in the same way for both groups.

Table 11 and Table 12 show that value-positive residents have healthier lifestyles and eating behaviors than hedonic residents. This is consistent with the literature, which posits that values influence both behavior and lifestyle. For example, value-positive residents embrace a food-related lifestyle that is significantly different from value-negative and hedonic residents. The following statements as shown in Table 11 manifestly define value-positive residents’ food-related lifestyle. These are: I prefer to buy fresh meat and vegetables rather than pre-packed, I try to avoid food products with additives, it is more important to choose food products for their nutritional value rather than for their taste, I like to have ample time in the kitchen and I prefer to buy low-fat food products, product information is of high importance, I need to know what the product contains, and I compare labels to select the most nutritious foods.

As shown in Table 11, value-positive residents display healthier behaviors when compared with value-negative and hedonic residents. They read the labels on food products, eat green salad, eat fish, eat fruits, and eat lean meats and lentils, which is consistent with their preferred lifestyle. In contrast, hedonic residents eat sauces, creams, and butter, use ready prepared dishes, eat more than one course at dinner, eat lunch, and dine at restaurants or café more frequently. These food-related behaviors are less supportive of a healthy lifestyle.

## 4. Conclusions

The results show that residents accorded different levels of importance to the underlying dimensions of food values derived from factor analysis. Knowledge of these differences will enable policy makers, program planners, and educators to select more effective strategies for implementing policies and programs in support of the adoption of healthier eating habits. For example, the higher level of importance accorded to taste suggests the need to emphasize palatability in preparing healthier meals, especially for older low-income individuals. The gender differences suggest that a different approach is necessary for encouraging males who have a negative view of food values to adjust their eating habits—the one size fits all approach will be ineffective. For males as well as the hedonic segment, intervention programs should take advantage of social learning and the network effect to advance the adoption of healthier eating habits. This is because behaviors are shaped by the stream of ideas to which individuals are exposed [17]. Additionally, for the hedonic segment, intervention programs should graft the desirable behavior to existing behaviors. For example, since residents in the hedonic segment like to eat out, they could be encouraged to select healthier offerings from the menu or select restaurants that offer healthier menu selections. This is a strategy for meeting residents where they are, which may prove more fruitful than badgering them with pedantic lessons about why they need to make changes to their food-related lifestyle and behavior.

The results provide yet another avenue for addressing the adoption of healthier eating habits. For example, the 47.5% of residents who make up the value-positive segments could serve as model individuals who interact with selected target individuals whose health behavior is the object of change (this is a slight modification of the [17] buddy group arrangement). However, the essential idea is similar. The goal is to create exposure to the positive examples of behavior of peers and cultivate a level of engagement around the issues that creates social pressure and nudges behavior of the target in the right direction. Further, an information-provision tool emphasizing value-based education should be tailored to strengthen social food values, especially for hedonic and value-negative residents in the study area, through active or participatory learning. Given the findings of the study, public health professionals should strive to implement a health promotion campaign aimed at improving consumers’ food values. Increasing awareness of consumers on fairness, tradition, origin, nutrition, naturalness, and environmental impact will raise consumers’ health consciousness, which, in turn, will lead consumers towards ethical and environmentally friendly foods that can make them more likely to eat healthy foods in the long term.

The data in this study provide program designers and policy makers with the opportunity to go beyond access in addressing the negative impacts of poor dietary behavior, and instead take advantage of the opportunities to design intervention programs based on the analysis that revealed food-related values as key driver of the system.

## Figures and Tables

**Table 1:** Descriptive statistics and importance ranking for food values.

Food Values	Mean *	Standard Deviation	Score	%	Rank
Naturalness	8.26	2.11	2907	82.59	6
Taste	9.39	1.31	3306	93.92	2
Price	7.99	2.46	2812	79.89	7
Safety	9.42	1.38	3315	94.18	1
Convenience	7.09	2.79	2494	70.85	9
Nutrition	8.82	1.57	3106	88.24	3
Tradition	5.77	2.98	2030	57.67	11
Origin	7.15	2.70	2518	71.53	8
Fairness	6.80	3.20	2393	67.98	10
Appearance	8.67	1.82	3052	86.70	4
Environmental Impact	8.29	2.07	2918	82.90	5

* 1: Not at all important, 10: Very important; Null hypothesis was rejected under Friedman Test for *p* < 0.01; Cronbach’s Alpha: 0.78.

**Table 2:** Comparative Importance of Food Values in the Literature.

Authors	Country	The Most	Intermediate	The Least
Lusk and Briggeman (2009) [60].	USA	Safety, nutrition, taste, price	Convenience, appearance, naturalness	Environmental impact, fairness, tradition, origin
Bazzani et al. (2016) [61].	USA	Safety, price, taste, nutrition	Naturalness, animal welfare, environmental impact, fairness	Appearance, origin, convenience, novelty
Present study	USA	Safety, taste, nutrition, appearance	Environmental impact, naturalness, price	Origin, convenience, fairness, tradition

**Table 3:** Summary of factor analysis results for food values of consumers.

Value Dimension	Mean *	Standard Deviation	Factor Loading
Ethical			
Fairness	6.80	3.20	0.890
Tradition	5.77	2.98	0.883
Origin	7.15	2.70	0.666
Social and Environmental			
Nutrition	8.82	1.57	0.819
Naturalness	8.26	2.11	0.651
Environmental Impact	8.29	2.07	0.645
Sensory (emotional)			
Appearance	8.67	1.82	0.776
Taste	9.39	1.31	0.766
Total variance explained (%)			64.554
Kaiser-Meyer-Olkin measure of sampling adequacy			0.720
Bartlett’s test of sphericity			638.668
Sig.			0.000
Cronbach’s Alpha			0.738

* 1: Not at all important, 10: Very important.

**Table 4:** Comparison between value dimensions.

Food Values	Mean *	Standard Deviation	Chi-Square	Asymp. Sig.
Ethical	6.57	2.50	253.291	0.000
Social and Environmental	8.46	1.42		
Sensory (emotional)	9.03	1.26		

* 1: Not at all important, 10: Very important; Null hypothesis was rejected under Friedman Test for *p* < 0.01.

**Table 5:** Resident segments.

Food Values	Segment I (Value-Positive)	Segment II (Value-Negative)	Segment III (Hedonic)	Kruskal-Wallis H	Asymp. Sig. *
Ethical	0.69793	−0.45086	−0.74148	166.20	0.00
Social and Environmental	0.37871	−0.08390	−0.49225	39.76	0.00
Sensory (emotional)	0.21130	−1.54011	0.56059	167.48	0.00
Number of respondents	168	66	118		
% of respondents	47.70	18.80	33.50		

* Null hypothesis was rejected under Kruskal Wallis test for *p* < 0.01.

**Table 6:** Demographic variables by segments.

Variables	Value-Positive	Value-Negative	Hedonic	Kruskal-Wallis H	Asymp. Sig. ***
Age	62.28 ^a^	57.23 ^b^	54.50 ^b^	19.757	0.000
Income *	3.19	3.35	3.26	2.03	0.362
Education **	2.37 ^a^	2.80 ^b^	2.73 ^b^	10.465	0.005

* 1: Less than $10 K, 4: $40 K+; ** 1: High school or below, 4: Postgraduate; *** Null hypothesis was rejected under Kruskal Wallis test for *p* < 0.01. ^a, b^ Different letters indicate differences between segments.

**Table 7:** The details of the number and percentage of demographic variables.

Group	Food Desert		Non-Food Desert		Total
	Count	%	Count	%	Count
**Gender**					
Male	34	41.46	94	35.07	128
Female	48	58.54	174	64.93	222
Age					
Less than 25	4	4.88	5	1.87	9
26–35	3	3.66	19	7.09	22
36–45	14	17.07	36	13.43	50
46–55	17	20.73	47	17.54	64
Greater than 56	44	53.66	161	60.07	205
Income					
Less than 10 K	5	6.1	12	4.48	17
11 K–25 K	16	19.51	37	13.81	53
26 K–40 K	25	30.49	72	26.87	97
Greater than 41 K	33	40.24	138	51.49	171
N/A	3	3.66	9	3.36	12
Education					
High school	22	26.8	68	25.37	90
Associate	16	19.15	31	11.57	47
College	29	35.37	107	39.93	136
Postgraduate					
Employment Status					
Unemployed	12	39.02	119	44.24	151
Part time	5	6.1	11	4.09	16
Full time	45	54.88	138	51.3	183
Total	82		268	268	350

**Table 8:** Perception of underlying dimensions by gender.

Food Values	Female	Male	Mann-Whitney U	Asymp. Sig.
Ethical	0.03088	−0.05337	13,611	0.401
Social and Environmental^*^	0.09431	−0.16304	12,505	0.041
Sensory (emotional)^**^	0.07987	−0.13806	12,675	0.063

^*, **^ Null hypothesis was rejected under Mann-Whitney U test for *p* < 0.10.

**Table 9:** Perception of underlying dimensions by food desert and non-food desert.

Food Values	Food Desert	Non-Food Desert	Mann-Whitney U	Asymp. Sig.
Ethical *	0.35138	−0.10019	8516.5	0.003
Social and Environmental	−0.09428	0.04192	10,437.5	0.567
Sensory (emotional)	−0.04600	0.00748	9645.5	0.118

* Null hypothesis was rejected under Mann-Whitney U test for *p* < 0.01.

**Table 10:** Food desert and non-food desert by consumer segments.

		Value-Positive	Value-Negative	Hedonic	Total
Food Desert	Count	44	13	24	81
	% within Food Desert area or not	54.30%	16.00%	29.60%	100.00%
	% within Cluster	26.20%	19.70%	20.70%	23.10%
	% of Total	12.60%	3.70%	6.90%	23.10%
Non Food Desert	Count	124	53	92	269
	% within Food Desert area or not	46.10%	19.70%	34.20%	100.00%
	% within Cluster	73.80%	80.30%	79.30%	76.90%
	% of Total	35.40%	15.10%	26.30%	76.90%
Total	Count	168	66	116	350
	% within Food Desert area or not	48.00%	18.90%	33.10%	100.00%
	% within Cluster	100.00%	100.00%	100.00%	100.00%
	% of Total	48.00%	18.90%	33.10%	100.00%

**Table 11:** Food-related lifestyle by consumer segments.

Lifestyle^*^	Value-Positive	Value-Negative	Hedonic	Kruskal-Wallis H	Asymp. Sig.^**^
The product information is of high importance. I need to know what the product contains.	6.02	5.41	5.31	14.717	0.001
I find taste in food products important	6.28	5.56	6.53	25.849	0.000
It is important for me to know that I get quality food for all my money	6.34	6.11	6.54	4.300	0.117
I eat before I get hungry, which means that I am never hungry at mealtimes	3.17	2.86	2.56	7.301	0.026
I compare product information labels to decide which brand to buy	5.03	4.79	4.26	8.438	0.015
I compare prices between product variants in order to get the best value for my money	5.92	5.38	5.89	4.717	0.095
We use a lot of ready-to-eat foods in our household	3.35	3.35	3.53	0.578	0.749
I notice when products I buy regularly change in price	5.87	4.92	5.41	6.326	0.042
I don’t like spending too much time on cooking	4.16	3.94	4.64	5.661	0.059
When cooking I first and foremost consider taste	5.27	5.15	5.82	9.188	0.010
I prefer fresh products than canned or frozen products	6.13	5.60	5.32	18.532	0.000
Snacking has taken over and replaced set eating hours	2.96	2.65	2.33	10.257	0.006
Going out for dinner is a regular part of our eating habits	4.05	3.70	4.84	15.088	0.001
I look for ads in the newspaper for store specials and plan to take advantage of them when I go shopping	4.50	3.70	4.11	6.176	0.046
I compare labels to select the most nutritious food	5.05	4.59	4.18	14.317	0.001
The wholesomeness of the food that I buy is an important quality	5.74	5.24	5.22	7.343	0.025
I do not buy food products that do not seem entirely fresh	5.94	5.85	6.15	0.986	0.611
I always check prices, even on small items	5.87	4.83	5.39	13.663	0.001
I enjoy going to restaurants with my family and friends	5.31	4.97	5.54	2.277	0.320
I like to have ample time in the kitchen	4.74	4.03	3.74	14.040	0.001
We often get together with friends to enjoy an easy-to-cook, casual dinner	3.95	3.52	4.05	3.818	0.148
I prefer to buy fresh meat and vegetables rather than prepacked	5.95	5.32	5.30	10.483	0.005
I try to avoid food products with additives.	5.21	4.39	4.26	17.200	0.000
It is more important to choose food products for their nutritional value rather than for their taste	5.17	4.75	4.40	14.118	0.001
Frozen foods account for a large part of the food products we use in our household	4.07	3.88	3.63	3.582	0.167
I prefer to buy low-fat food products	4.27	3.82	3.26	15.709	0.000
I use a lot of instant mixes such as baking mixes and powder soups	2.77	2.53	3.35	5.632	0.060
Cooking is a task	4.02	4.56	4.78	8.798	0.012
I always try to get the best quality for the best price	6.01	5.65	6.26	6.437	0.040
I eat whenever I feel the slightest bit hungry	3.01	2.67	2.57	5.322	0.070

* 1: Completely disagree, 7: Completely agree; ** Null hypothesis was rejected under Kruskal Wallis test for *p* < 0.10.

**Table 12:** Food-related behaviors by consumer segments.

Behaviors^*^	Value-Positive	Value-Negative	Hedonic	Kruskal-Wallis H	Asymp. Sig.^**^
I read the informative labels on the food products in the supermarket	3.61	3.55	3.25	6.066	0.048
I buy food products at the supermarket	4.30	4.33	4.23	1.598	0.450
I eat green salad	4.51	4.14	3.91	30.294	0.000
I eat fish	4.36	3.41	3.51	47.629	0.000
I eat fruit	4.50	4.11	4.03	21.954	0.000
I eat lentils	3.29	2.69	2.81	12.321	0.002
I eat lean meat	4.17	3.81	3.92	8.138	0.017
I eat sauces with cream and butter in my food	3.20	2.59	3.34	17.732	0.000
I eat sweets and cakes	3.19	2.77	3.02	4.874	0.087
I buy organic food products	2.50	2.38	2.30	1.377	0.502
I use ready-prepared dishes that just need to be heated up	2.32	2.39	2.60	6.261	0.044
I eat more than one course at dinner	2.96	2.76	3.31	9.460	0.009
I snack instead of eating a big dinner	2.23	2.12	2.11	0.595	0.743
I eat lunch/dine at a café/restaurant	2.67	2.88	3.24	16.098	0.000
I eat lunch/dine with family/friends	3.00	3.08	3.35	6.355	0.042
I eat fast food	2.71	2.71	3.00	7.676	0.022

* 1: Never, 5: Always; * Null hypothesis was rejected under Kruskal Wallis test for *p* < 0.10.

## Data Availability

The data presented in this study are available on request from the corresponding author.
